# Pathogenesis of preterm birth: bidirectional inflammation in mother and fetus

**DOI:** 10.1007/s00281-020-00807-y

**Published:** 2020-09-07

**Authors:** Ella Shana Green, Petra Clara Arck

**Affiliations:** Department of Obstetrics and Fetal Medicine, Laboratory for Experimental Feto-Maternal Medicine, University Medical Center Hamburg-Eppendorf, Martinistraße 52, 20251 Hamburg, Germany

**Keywords:** Preterm birth, Labor, Mouse models, Regulatory T cells, Inflammatory signaling pathways, Microbiome, Fetal signals

## Abstract

Preterm birth (PTB) complicates 5–18% of pregnancies globally and is a leading cause of maternal and fetal morbidity and mortality. Most PTB is spontaneous and idiopathic, with largely undefined causes. To increase understanding of PTB, much research in recent years has focused on using animal models to recapitulate the pathophysiology of PTB. Dysfunctions of maternal immune adaptations have been implicated in a range of pregnancy pathologies, including PTB. A wealth of evidence arising from mouse models as well as human studies is now available to support that PTB results from a breakdown in fetal-maternal tolerance, along with excessive, premature inflammation. In this review, we examine the current knowledge of the bidirectional communication between fetal and maternal systems and its role in the immunopathogenesis of PTB. These recent insights significantly advance our understanding of the pathogenesis of PTB, which is essential to ultimately designing more effective strategies for early prediction and subsequent prevention of PTB.

## Introduction

Preterm birth (PTB), defined as birth before the 37th week of gestation, affects up to 15 million pregnancies each year globally [1]. Being born too soon can lead to neonatal death, but also a high risk for early-life infections and neurodevelopmental, cardiometabolic, and inflammatory disorders later in the life of surviving infants [2–6]. A current understanding of the complex pathogenesis of PTB is still poor, which also explains the limited availability of targeted and effective strategies for PTB prevention.

Several risk factors for PTB are well recognized, such as twin pregnancies, chorioamnionitis, pre-existing maternal diseases, genetic factors, previous PTB, and uterine abnormalities [7–10]. However, a large proportion of PTBs have no identifiable cause. These spontaneous PTBs present as pathological inflammation, premature rupture of membranes (PROM), and onset of preterm labor.

Interestingly, inflammation is also a key trigger of the physiological onset of labor at term [11]. Thus, the onset of inflammation seems to be a common denominator initiating term as well as preterm labor. Here, we comprehensively review why inflammation may be prematurely initiated in some pregnancies, which then leads to PTB.

## A brief overview of maternal immune adaptation during normally progressing pregnancies

**Fig. 1:**
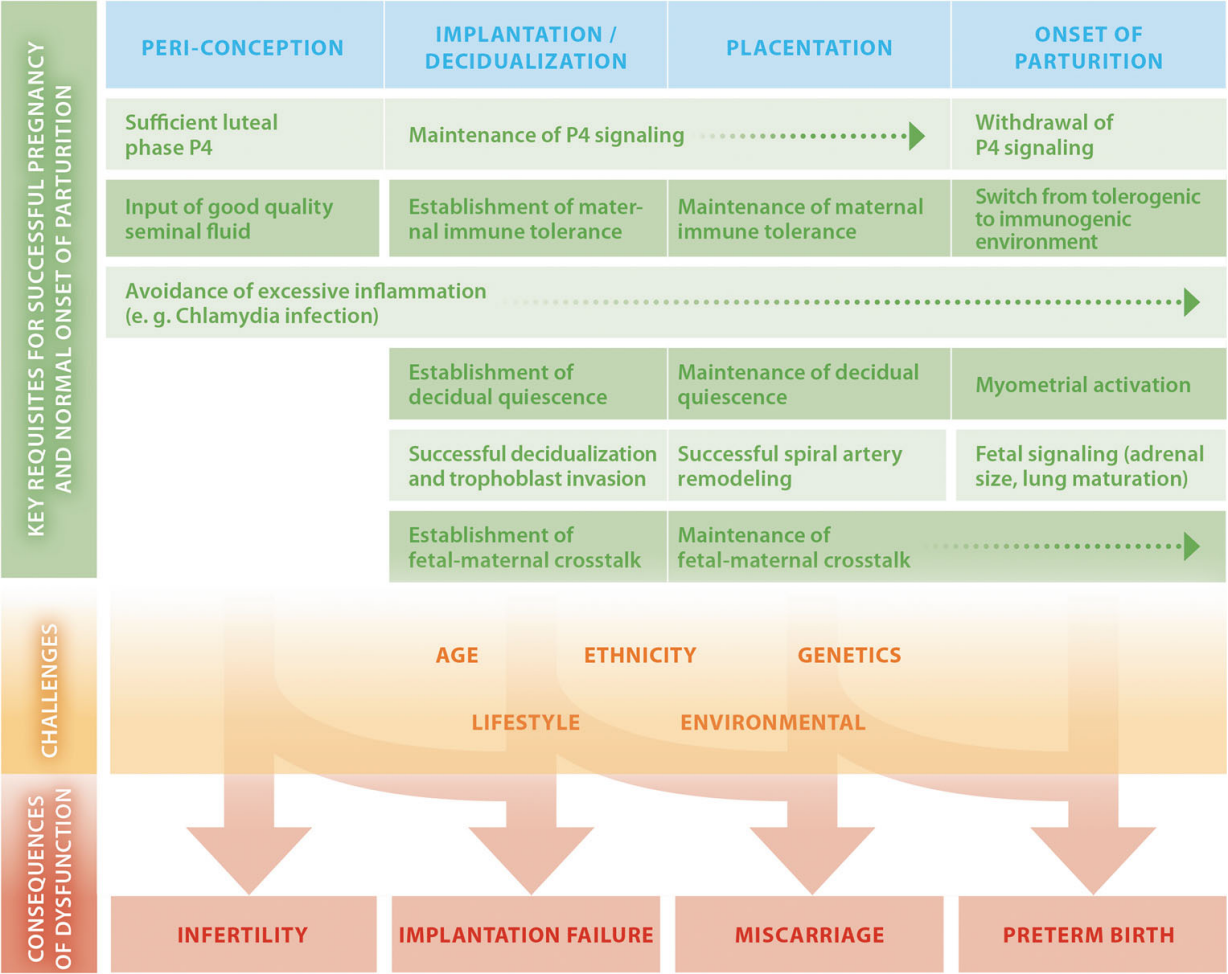
Milestones of pregnancy and related immune and endocrine adaptations. Normally progressing pregnancies result from the successful orchestration of processes involved in peri-conception, implantation, decidualization, and placentation. Hormone signaling, in particular, progesterone (P4), establishes a receptive endometrium and decidual quiescence; maternal immune tolerance mitigates the risk for fetal rejection and supports spiral artery remodeling and placentation. The onset of parturition is characterized by a switch in the local immune environment from a tolerogenic state to an activation state, a reversal of decidual quiescence in favor of myometrial activation, and a withdrawal of P4 signaling. Maternal and paternal lifestyle factors (such as stress and nutrition), environmental factors, age, ethnicity, and genetics contribute to the success or failure of pregnancy, including the risk for PTB [9, 12, 13]

In order to understand the onset of inflammation during pregnancy, it is pivotal to summarize milestones of pregnancy (Fig. 1), including key features of feto-maternal immune tolerance, as the breakdown of such immune tolerance is associated with a number of pregnancy complications [14–17].

As covered by a large number of reviews [14, 15, 17–20], the need for feto-maternal immune tolerance arises from the expression of paternal antigens on fetal tissues. The maternal immune system recognizes and responds to these fetal/paternal antigens firstly at coitus, when the reproductive tract comes into contact with seminal fluid, followed by continuous paternal antigen contact throughout pregnancy upon the invasion of fetal trophoblast cells into the endometrium. Notably, the process of implantation itself is an inflammatory process, during which cytokines, chemokines, and prostaglandins contained in seminal fluid elicit pro-inflammatory cytokine production and leukocyte recruitment within the uterus [14]. Around the time of implantation, this inflammation needs to be resolved and a tolerogenic environment induced. Numerous mechanisms are mounted in the induction of such tolerogenic environment, including the release of anti-inflammatory molecules such as TGFβ [21] and the induction of specialized, anti-inflammatory T cells known as CD4^+^FOXP3^+^ regulatory T (Treg) cells, which constrain anti-fetal inflammatory immune responses [22–26]. Tolerogenic dendritic cells (DCs) uniquely present in the decidua cross-present fetal antigens to maternal CD4^+^ T cells, thereby also activating the generation of Treg cells [27]. Treg cells reciprocally interact with DCs and macrophages, modifying their phenotypic states to be pro-tolerogenic [28]. Importantly, Treg cells protect against anti-fetal immune responses by limiting existing T effector (Teff) cell responses and maintaining anergy in the T conventional (Tcon) cell population that would otherwise become Teff cells [25, 29]. Placental cells also contribute to the generation of a tolerogenic environment, e.g., via trophoblast-derived colony-stimulating factor (CSF)1 (formerly M-CSF) and interleukin (IL)10, which induces decidual macrophages with a regulatory “M2” phenotype, expands functionally suppressive CD4^+^FOXP3^+^ Treg cells, and limits activation of T helper (Th)1-, Th17-, and Th2-cytokine-producing Teff cells [30].

## Maternal-fetal crosstalk and reproductive success

In normal pregnancy, there is extensive crosstalk between maternal and fetal systems due to the sharing of circulatory systems in the placenta. Nutrients and metabolic waste products are exchanged from mother to fetus and vice versa, along with cells, signaling molecules, extracellular vesicles (ECVs), and nucleic acids. The exchange of cells results in microchimerism in mother or fetus, whereby maternal cells transferred to the fetus are referred to as maternal microchimerism (MMC). Reciprocally, fetal cells transferred to the mother are termed fetal microchimerism (FMC). Both, MMC and FMC can persist for decades after pregnancy in a broad range of tissues in the body, including the brain, skin, heart, lung, bone marrow, spleen, and lymph nodes [31–34].

MMC cells promote tolerance to maternal antigens in fetal immune cells during pregnancy through the induction of non-inherited maternal antigen (NIMA)–specific CD4^+^FOXP3^+^ Treg cells [35]. Furthermore, they may enhance reproductive fitness in the next generation through sustained tolerance to NIMA. However, these insights are solely based on a mouse model to date and limited to settings where the paternal antigens and NIMA were shared [36].

In addition, microvesicles, exosomes, and cell-free (cf) proteins and nucleic acids such and cfRNA and cfDNA are exchanged via the placenta. In general, the concentration of cfRNA in maternal circulation increases over the course of gestation [37, 38], and fetal cfDNA and RNA transcripts also increase in maternal circulation late in gestation [39–42]. Some fetal cfDNA and RNA are contained within apoptotic fetal cells [39, 41], but immunosuppressive CD71^+^ fetal erythroid cells are also an abundant source of fetal DNA in maternal circulation [37]. The cfDNA and RNA transcripts could be transferred passively, or via ECVs. Exosomes are a type of ECV released by exocytosis into the extracellular environment. Notably, the placenta releases exosomes into maternal circulation during pregnancy [43], which increase with increasing gestational age peaking at term [44, 45]. The functional importance of various ECVs in pregnancy is largely unknown, but they are suspected to mediate fetal-maternal communication at key stages of pregnancy including implantation and parturition [43, 46]. Human villous trophoblasts secrete exosomes containing placenta-specific miRNAs into maternal circulation, which play key roles in regulating immune signaling [47, 48]. Maternal-derived exosomes are also enriched during pregnancy, and the transfer of exosomes between the fetus and mother is bidirectional [49].

## Immune tolerance versus inflammation in the development of preterm birth

As mentioned above, inflammation has been linked to the pathogenesis of idiopathic PTB, and also other pregnancy complications in mice and humans [7, 50–55]. Furthermore, PTB is considered to have early pregnancy origins, prior to placental development. Thus, it has been proposed that PTB is a disorder that results from defective deep placentation [56, 57], similar to preeclampsia.

**Fig. 2:**
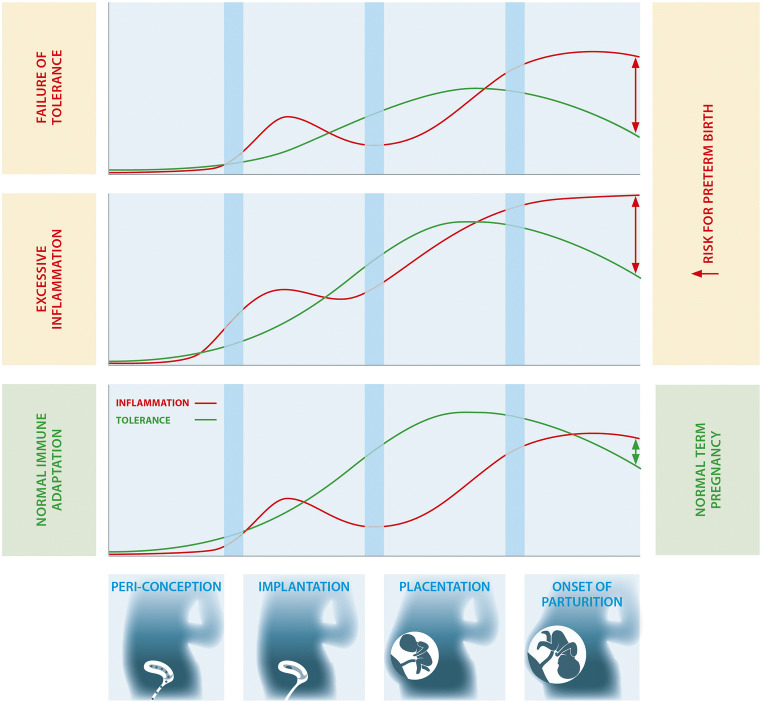
Trajectories of maternal immune tolerance versus inflammation determines the susceptibility to PTB. A balanced tolerogenic and inflammatory immune response underlies the normal progressing of pregnancy and onset of birth at term. Failure to mount sufficient levels of tolerance (top panel) or excess inflammation (middle panel) creates an immune imbalance in favor of inflammation, thereby increasing the risk for PTB

As the maternal immune adaptation is involved in the processes sustaining pregnancy success throughout the entire period of gestation, poor immune adaptation from the outset of pregnancy and the failure to mount immune tolerance or to dampen excessive inflammation could be implicated in the cause of PTB (Fig. 2). This theory extends the possibility that early immune perturbations can have a lasting, detrimental effect on the pregnancy, as proposed in [17].

Early immune perturbations caused by maternal stress perception, infection, and diet, may result in blunted establishment of immune tolerance and excess inflammation [14, 16, 17, 58, 59]. In addition, the vaginal microbiota composition may be of relevance, as a differential vaginal microbial composition has been associated with an increased risk of PTB in women with African ancestry [60, 61].

Challenges occurring later during gestation, such as maternal infection, endocrine dysfunction, microbiota composition, or metabolic dysregulation, can also further exacerbate the inflammatory burden and lead to PTB [7, 10, 62, 63].

## Immune cells involved in term and preterm labor

Considering the importance of inflammation in the onset of labor at term or preterm, it is key to understand the mechanisms behind its origin. In this context, mouse models have become useful tools to provide insights into the complexity of PTB (Table 1). There are various ways to induce preterm labor (PTL) and birth in mice, including immune, hormonal, and environmental agents. Immunomodulatory agents activate or exacerbate the physiologically occurring inflammatory pathways involved in labor induction and recapitulate the clinical manifestations of labor associated with infection and/or inflammation (Table 1). The most common mouse models of PTB involve the administration of lipopolysaccharide (LPS), a bacterial cell wall component of gram-negative bacteria, or whole heat-killed bacteria such as *Escherichia coli* (*E. coli*) via intrauterine or intraperitoneal routes of administration. Importantly, correlations made between these studies are limited due to differences in dosage, route, and timing of the compound administered, as well as the outcomes measured. For an extensive list of other models of PTB, we refer to the recent study by McCarthy et al. [88]. The use of mouse strains with specific genetic mutations has further revealed the importance of numerous proteins and signaling pathways in normal and/or preterm labor (Table 1). However, most of these studies carry certain limitations and care should be taken to interpret the data. Firstly, the use of global knockout mice does not allow for analysis of the effect of cell-specific mutations and can complicate interpretations if littermate controls are not used and if pre-pregnancy immune and endocrine status is not considered. Furthermore, as most studies also mate to the same genotype male, maternal versus fetal effects can be difficult to determine. Finally, the background strain of the mutant mice used is important to note, since genetic background is a determinant of gestation length in mice [89]. Despite their limitations, studies using either interventions or mutant mouse lines to cause or delay preterm or term labor provide valuable insights into the inflammatory mechanisms of these processes and highlight the balance of factors that work in concert to produce controlled inflammation in normal term labor. Importantly, dysregulation of many essential components of these inflammatory pathways either prevents or increases susceptibility to preterm labor in mice.

**Table 1:** Interventions and mutant mouse strains used to study the pathophysiology of preterm birth

Interventions used to induce PTL in wild-type mice	
Lipopolysaccharide (LPS)	[63–70]
*E. coli*	[71]
Poly(I:C)	[63]
IL1β	[72]
Anti-CD3e	[73]
Anti-galactosylceramide (a-GalCer)	[74]
Plasma exosomes	[75]
Recombinant IL6	[65]
In vitro activated neonatal CD4^+^ T cells	[53]
HMGB1	[76]
IL10 neutralizing antibody	[67]
Low dose influenza virus (PR8)	[63]
Lymphocytic choriomeningitis virus (LCMV)	[63]
Interventions used to prevent PTL in wild-type mice	
Anti-F480	[77]
(+)-Naloxone (toll-like receptor-4 antagonist)	[78]
101.10 (IL-1R-biased ligand)	[64]
Mutant mouse strains used to study term and preterm labor	
Recombination activating gene (Rag) 1	[79]
Interleukin 6 (IL6)	[65]
Type I IFN receptor	[63, 64]
Myeloid differentiation primary-response gene 88 (MyD88)	[71]
TIR domain-containing adaptor protein-inducing IFNβ (TRIF)	[71]
Myd88 and TRIF	[71]
PAF acetylhydrolase (PAF-AH)	[80]
Complement (anaphylatoxin C5a)	[77]
Steroid receptor coactivase 1 (SRC-1)	[81]
Steroid receptor coactivase 2 (SRC-2)	[81]
Heterozygous mutation in SRC-1 and 2	[81]
Toll-like receptor 3 (TLR2)	[82]
Surfactant protein (SP)-A	[82]
Surfactant protein (SP)-D	[82]
SP-A and SP-D	[82]
CXC3 chemokine receptor 1 (CXC3CR1)	[83]
Toll-like receptor 4 (TLR4)	[70]
TLR4 deletion in progesterone receptor (Pgr)–expressing cells	[84]
TLR4 deletion in Tie2-expressing cells	[84]
p53 deletion in progesterone receptor (Pgr)–expressing cells	[84]
Trp53 deletion in Pgr-expressing cells	[85, 86]
Prostaglandin F2 alpha receptor	[87]
Oxytocin and PGF2α receptor	[87]
Oxytocin receptor and PGF2α receptor	[87]

Using mouse models of PTB, macrophages were identified as major contributors to the induction of term and preterm labor (PTL). Depletion of macrophages using anti-F4/80 administration prior to experimental LPS-induced onset of PTB eliminates susceptibility to PTB [51]. Macrophages likely contribute to the pathogenesis of PTB via secretion of inflammatory cytokines, such as tumor necrosis factor (TNF)α, IL1, IL6, and IL8 and uterine contractility genes such as matrix metalloproteinases (MMPs) [90]. In particular, IL1 is known to critically contribute to inflammation-induced PTB. As shown in mice, IL1 administration induces PTL, and inhibition of its receptor prevents labor [72], likely via activation of NF-κb signaling pathways [64]. Similarly, IL6 is another cytokine important in the timing of parturition and the pathogenesis of PTB, since IL6-deficient mice have delayed parturition and are also resistant to LPS-induced PTB [65].

Moreover, complement activation plays an important role in PTB. This has been observed in infection-induced PTB in humans, where women with spontaneous PTL showed increased concentrations of complement products C3a, C4a, and C5a [51]. In mice, complement receptor C5aR-deficient mice are protected against LPS- and RU486-induced PTB. In this study, increased complement deposition was present in the cervical epithelium of PTB mice, along with complement C5a activation–dependent MMP9 release from macrophages and cervical remodeling [77].

Besides these examples of the involvement of the innate immune system in modulating the risk for PTB, the adaptive immune response is also critical. A subset of PTB is postulated to result from a failure of maternal tolerance to fetal antigens [7]. Indeed, Treg and Teff cells exhibit distinct changes in frequency and phenotype in PTB. Treg cells in peripheral blood of women in PTL are reported to have differential activation and diminished suppressive capacity compared with term controls [52, 91, 92]. Normal human labor is associated with a decline in Treg cell function and reciprocal activation of Teff cells which are normally controlled by Treg cells [92].

A decrease in Treg cell number and/or function in pregnancy pathologies is typically associated with an increase in Tcon/Teff activation. This holds true for labor, as T cells with an activated memory phenotype are increased in the choriodecidua (fetal membranes) of women with spontaneous labor at term [93]. Effector memory CD4^+^ and CD8^+^ T cells are also enriched in the decidua in spontaneous PTL, suggesting the inflammatory potential of these cells is greater in PTL women [73]. CD4^+^ and CD8^+^ T cells expressing “exhausted” (PD-1^+^TIM-3^+^CTLA4^−^LAG-3^−^) and “senescent” (KLRG-1^+^CD57^+^) memory and effector phenotypes were identified in the decidua of women with term and preterm labor [66]. Notably, the proportions of exhausted CD4^+^ T cells increased in the decidua parietalis with increasing gestational age but declined in the decidua basalis of women who underwent PTL with placental inflammation. As TNFα and interferon (IFN)γ production could be induced *ex vivo* in the exhausted T cells, these cells may restore their effector function upon placental inflammation [66].

T and B cell–deficient Rag1^−/−^ mice have provided important clues for the role of T cells in PTB. These mice show an increased susceptibility to LPS-induced PTB, which may be mediated by macrophage activation. However, adoptive transfer of CD4^+^ T cells at mid-gestation conferred resistance to LPS-induced PTB, as these cells seem to rapidly differentiate into Treg cells [79]. Conversely, activation of Teff cells using anti-CD3 causes PTB via upregulation of local and systemic pro-inflammatory responses such as IL6 and IFNγ [73]. Interestingly, in utero adoptive transfer of CD4^+^ and CD8^+^ Teff cells causes late fetal resorption dependent on TNFα and IFNγ. These cytokines also cause uterine contractility in vitro. However, it is unclear whether these Teff cells cause PTB if administered later or systemically [54].

Together, these studies suggest that activated memory T cells and Teff cells may participate in mediating inflammatory processes in normal term labor, whereas Treg cells may act to minimize the inflammatory potential or premature activation of exhausted and memory Teff cells. However, the molecular details of these maternal T cell interactions implicated in normal and PTL are yet to be defined. One potential pathway could involve IL10, as Treg cells are capable of producing this anti-inflammatory cytokine. IL10 has been shown to prevent PTB, as IL10 deficiency or administration of IL10-neutralizing antibodies increases susceptibility to PTB in mice [67]. As global IL10 deficiency led to a decrease in inflammatory cytokine gene expression in the uterus and placenta, IL10 may act to regulate excessive inflammatory responses implicated in labor. Recent work demonstrates that TLR4 signaling in decidual endothelial cells at term may cause IL10 induction in perivascular stromal cells via NF-κb activation of IL6 and STAT3. This could be a mechanism to preserve the homeostatic immune balance under inflammation, which may be perturbed in PTB [84].

Emerging data arising from humans and mice indicate that the fetal environment in PTB is also characterized by inflammation, along with the priming of fetal T cells against maternal antigens [54]. Future studies are now needed to confirm if fetal inflammation is causal to maternal inflammation and related PTB, or a consequence of the maternal spill-over of inflammatory cytokines.

NKT cells, an innate-like subset of T cells with specialized functions, are also implicated in PTB. Maternal NKT cells recognize CD1-restricted lipid antigens, which are expressed by fetal trophoblast cells, and are thought to play an immunoregulatory role in pregnancy [94]. Interestingly, depletion of invariant NKT cells reduces the rate of LPS-induced PTB in mice and administration of an antibody specific for NKT cells (α-GalCer) in late gestation causes PTB via activation of CD4^+^ T cells, macrophages, neutrophils, and DCs in the myometrium/decidua [68, 74, 95].

## Inflammatory signaling pathways in term and preterm labor

**Fig. 3:**
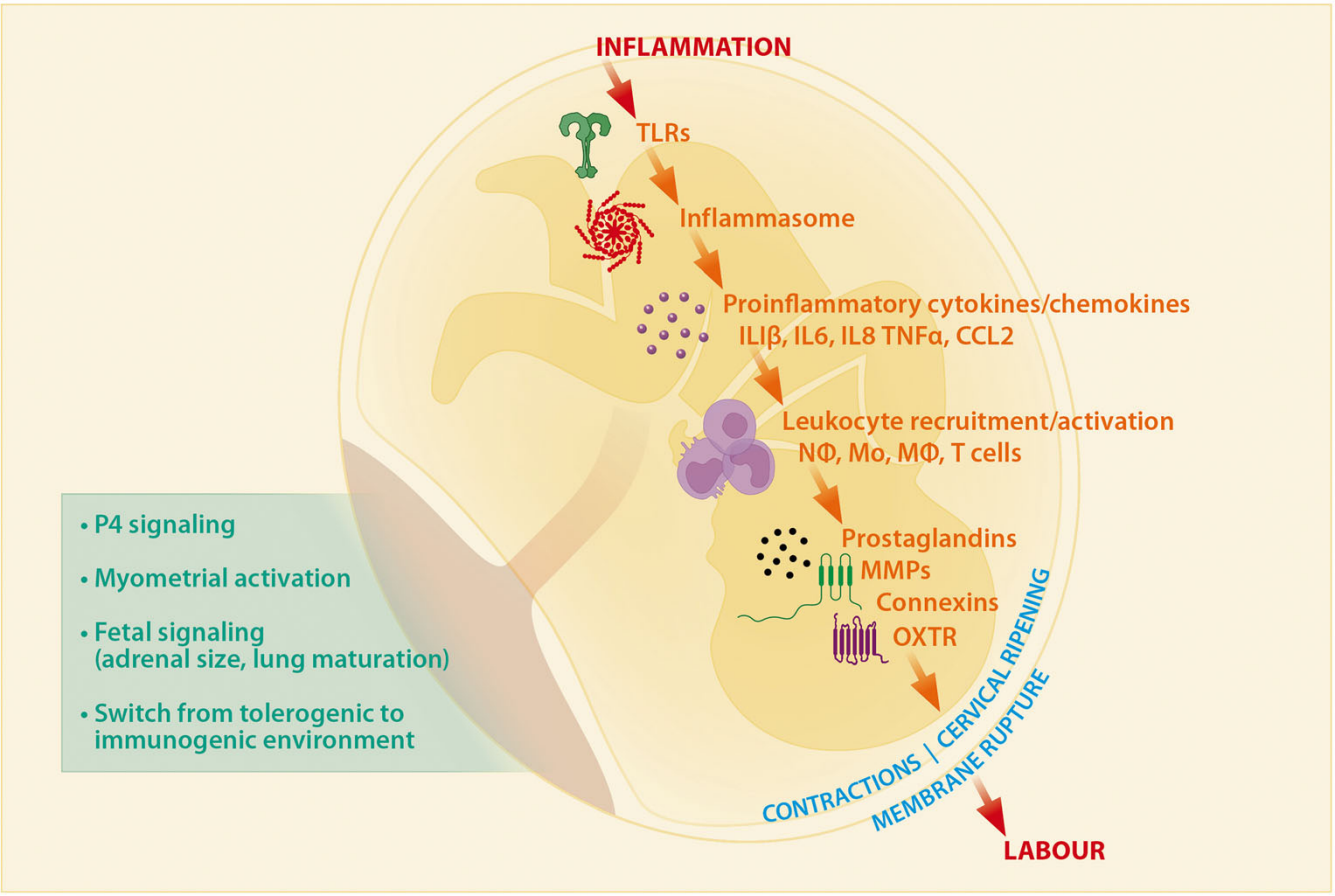
Current understanding of the inflammatory pathways of parturition. In pregnancy, toll-like receptors (TLRs) expressed on immune and endothelial cells at the fetal-maternal-interface are activated during sterile or active inflammation, via a range of DAMPs and PAMPs. TLR activation causes a cascade of intracellular signaling events leading to inflammasome activation and release of pro-inflammatory cytokines and chemokines. This leads to further recruitment of pro-inflammatory leukocytes such as macrophages, monocytes, neutrophils, and T cells to the decidua, placenta, and amniotic cavity. In macrophages, NF-κb activation controls expression of genes encoding uterine contractility and cervical ripening proteins such as prostaglandins F2α receptor, connexin-43, oxytocin receptor, and cyclooxygenase 2 (COX-2), as well as genes encoding pro-inflammatory cytokines TNFα, IL1β, IL6, and IL8. Uterine contractility and cervical ripening lead to membrane rupture and subsequently, the onset of labor. Withdrawal of P4 signaling, myometrial activation, fetal signals, and a switch in maternal immune phenotypes from tolerance to inflammation all contribute to the activation of the main inflammatory pathway. In the case of preterm birth, studies in mice show that various perturbations in this inflammatory pathway can occur, leading to premature activation of essential pathways components, resulting in preterm labor

A crucial component of inflammatory immune responses is the engagement of toll-like receptors (TLRs) which trigger signaling pathways leading to secretion of cytokines and chemokines by innate immune cells. TLRs are an evolutionarily conserved class of pattern recognition receptor (PRR) that recognize pathogen-associated molecular patterns (PAMPS) derived from microorganisms, and damage-associated molecular patterns (DAMPS) released from immune cells and stressed and dying cells. TLR activation is an essential initiating component of the inflammatory pathway that leads to both normal and preterm labor (Fig. 3).

Most research on the role of TLRs in PTB has focused on TLR4 which binds to a specific range of PAMP and DAMP ligands including bacterial LPS. TLR4 is activated in the uterus in both normal and PTB and is essential for on-time parturition in mice, controlling uterine activation and onset of labor [70]. Pregnant TLR4^−/−^ mice deliver an average of 13 h later than controls and their offspring exhibit reduced viability [70]. Furthermore, inhibition of TLR4 signaling with the TLR4-antagonist (+)-naloxone following intrauterine administration of *E. coli* suppressed the inflammatory cascade and effectively prevented against PTB [78]. Recently, TLR4 expression by decidual endothelial cells, and not immune cells, was identified to be a key for initiating this response, since mice with endothelial-specific TLR4 deletion are resistant to LPS-induced PTB [84]. In humans, maternal single nucleotide polymorphisms (SNPs) in the *TLR4* gene are associated with early preterm delivery before gestational week 32 [96]. Downstream of TLR4 and its coreceptor CD14 is MyD88-dependent and independent (TRIF-dependent) signaling pathways, that each control specific inflammatory gene expression. In mice, PTB is dependent on MyD88, since MyD88^−/−^ mice are completely protected against *E. coli*–induced PTB [71].

In addition to TLR4, other TLRs likely mediate timing of birth, such as TLR2 [82, 97]. In addition to maternal TLR expression, fetal TLR expression may have involvement in the timing of birth as polymorphic fetal TLR4 and TLR2 alleles were associated with prematurity [98, 99].

TLR activation subsequently leads to the activation of the inflammasome and the perpetuation of additional cytokine and chemokine production in the placenta and decidua, such as IL8, CCL2, IL1β, and IL6 [100]. TLR activation also initiates the recruitment of immune cells, production of prostaglandins, and MMPs, leading to the activation of cervical ripening and uterine contractions (Fig. 3). Also, the influx of immune cells such as macrophages to the amniotic cavity is pivotal for the onset of labor, as it induces the production of inflammatory mediators such as NF-κb. NF-κb is a key mediator of the inflammatory cascade leading to labor, since it directly binds to the promoters of genes that cause uterine contractility such as *PTGFR* (prostaglandin F2α receptor), *GJA1* (connexin 43), *OXTR* (oxytocin receptor), and *PTGS2* (cyclooxygenase 2; COX-2), as well as genes encoding pro-inflammatory cytokines TNFα, IL1β, IL6, and IL8 [90]. Taken together, PRRs, and specifically TLRs and their downstream signaling molecules, are key players in triggering the timing of birth at term and preterm.

The DAMPs and PAMPs which bind to PRRs also play essential roles as the “messengers” to initiate the inflammatory cascade leading to term and preterm labor. TLRs in pregnancy are activated during sterile inflammation, and in the absence of active infection, via DAMPs [101]. The most studied DAMPs in preterm and term labor are high-mobility group box 1 (HMGB1), fetal cfDNA, and platelet-activating factor (PAF). Oxidative stress and cellular senescence of the fetal amnion and chorion may trigger human parturition through the release of DAMPs. In senescent cells, DAMPs relocate from the nucleus to the cytosol where they can be secreted as alarmins and drive the inflammatory parturition cascade [102]. Intra-amniotic administration of HMGB1, an alarmin that induces inflammation upon tissue damage, causes preterm labor in mice [76] and is elevated in the amniotic fluid of women who underwent PTL, independent of intra-amniotic infection status [103]. HMGB1 was found to be primarily expressed by amnion epithelial cells, myofibroblasts, neutrophils, and macrophages [103], and incubation of chorioamniotic membranes with HMGB1 leads to release of pro-inflammatory IL1β and IL6 [104]. cfDNA, a common activator of TLRs, was found to be released upon cell death to cause TLR signaling in the placenta [105]. The inflammatory phospholipid PAF is another DAMP critical for labor. It is increased in the amniotic fluid of women who deliver preterm [106–108], and its administration induces PTB in mice via TLR signaling in macrophages [80, 108, 109]. Alarmins released from cells upon tissue stress can also bind to PRRs, and concentrations of alarmins IL1α and S11 family proteins calgranulin A and calgranulin C were increased in amniotic fluid during sterile intra-amniotic inflammation [110]. Other PRRs such as NRLP3 and NOD2 activate inflammasomes, which likely play a role in initiating spontaneous labor at term and preterm labor, since the activation of components of this pathway causes the secretion of IL1β in chorioamniotic membranes in normal labor at term [100]. Placental PRR expression mediates their functions as a key barrier capable of recognizing and responding to microbes and stress.

Perturbations affecting the placenta could therefore affect the placental response to PAMPs and DAMPs and have an impact on pregnancy outcome. In summary, DAMPs play key roles in initiating labor at term and are implicated in the cause of PTL; however, their origin (fetal versus maternal), triggers, and regulation are not well understood.

## Fetal-placental and vaginal microbiomes in PTB

Recent research endeavors aimed to identify the impact of the microbiome in modulating the risk for PTB [111]. Features of the vaginal microbiome have been shown to determine the risk for PTB. For example, in women of African ancestry, dramatic changes in the diversity of the microbiota were observed in early pregnancy, with an increased prevalence of multiple vagitypes and dysbiosis [61]. Several dysbiotic vagitypes could be associated with PTB, likely caused by an early pregnancy increase in pro-inflammatory cytokines in vaginal fluid, such as the chemoattractant CXCL10 [60]. Interestingly, preconception administration of antibiotics to women with previous preterm delivery did not reduce preterm birth rate and may be associated with earlier delivery and decreased birth weight [112], suggesting that complex host-microbiome dynamics from the outset of pregnancy are likely essential for optimal pregnancy success [113]. The molecular mechanisms of these interactions will be important to uncover in future research efforts. In contrast to the vaginal microbiota, there is still a great deal of ambiguity as to the question of whether the intrauterine environment is sterile or non-sterile. 16S rRNA sequencing and culture of bacterial species isolated from the female reproductive tract revealed microbial communities in the cervix, uterus, fallopian tubes, and peritoneal fluid that were diverse and distinct compared with the vagina and cervix, which were Lactobacillus dominated [114]. Examination of microbiota from the endometrial fluid of infertile women undergoing *in vitro* fertilization (IVF) revealed an association between non-Lactobacillus-dominated endometrial microbiota and decreases in implantation, pregnancy, and live birth rates [115]. Several mechanisms have been proposed to limit bacterial invasion to the uterus from the lower genital tract, and these may operate during pregnancy to ensure a tolerogenic environment is maintained for pregnancy success. Bacterial invasion of the uterus and amniotic cavity found in subsets of pregnant women could theoretically cause inflammation and disruption of the immune adaptation to pregnancy, predisposing to PTB [113]. Microorganisms similar to the vaginal microbiota can also be isolated from the placenta [116]. However, others have rejected this concept, showing contaminants were responsible for the detection of most bacterial species in the placenta [117]. Given these findings, there is still debate as to whether the uterus and placenta harbor a microbiome or not. Hence, it is still unknown if a skew of the intrauterine microbiome—if it indeed exists—may modulate the risk for PTB.

## The role of maternal progesterone in PTB

Hormone levels have also been extensively studied in order to understand the pathogenesis of PTB. Adequate progesterone (P4) levels are known to be essential for the establishment and progression of normal pregnancy. Hence, it is not surprising that labor is associated with a P4 withdrawal in mice and functional progesterone withdrawal in women [118, 119]. The P4 decline appears essential for labor to occur, as the administration of P4 to mice in late gestation extends the gestation length and prevents labor. Similarly, mice lacking the P4-metabolizing enzyme 20αHSD show prolonged gestation [120]. Conversely, treatment with the progesterone antagonist RU486 causes PTB in mice and is associated with increased decidual PGE2 and IL6 concentrations [121]. P4 application is also protective against LPS-induced PTB in mice via prevention of inflammation-induced cervical remodeling, cervical macrophage infiltration, and MMP9 expression that precedes PTB, possibly through the disruption of complement signaling on macrophages [77]. P4 is known as a strong immunomodulator, it induces stable Treg cells, arrests DC in a tolerogenic state, and suppresses inflammatory responses [122–124]. P4 signaling withdrawal may therefore be one mechanism of immune modulation leading to PTB. The P4 decline in late gestation may be triggered by immune pathways, as macrophages have been shown to regulate P4 production in the ovary in mice [125]. However, it should be noted that PTB in mice can also occur in the absence of P4 withdrawal, for instance, with intrauterine administration of bacteria [126]; multiple, independent immune and hormonal mechanisms are likely in place.

## Myometrial and decidual clocks in PTB

The decline in P4 signaling induces a switch of the myometrium from a quiescent to a contractile state at labor [127]. However, evidence places the establishment of myometrial quiescence also at the start of gestation, as it may be regulated by estrogen and P4 hormone receptor signaling [128]. Besides the myometrium, the endometrium and decidua are also proposed to be responsible for the timing of birth via induction of a decidual “clock.” This decidual clock mediates mechanisms of fetal-maternal tolerance in early pregnancy and wanes over time with advancing gestational age, leading to a withdrawal of active suppression or induction of inflammatory signals [10]. The induction of decidual quiescence is an active process occurring in early pregnancy, caused by transcriptional silencing in decidual stromal cells (DSCs) which recruit the repressive histone mark H3K27me3 for epigenetic regulation of hundreds of genes, causing suppression of type 1 immunity and wound healing response [129]. Again, P4 signaling from the outset of gestation may be essential to decidual quiescence and maintenance of an anti-inflammatory immune environment needed to last for the duration of pregnancy, until labor at term.

## Fetal origins of the timing of birth

There are several lines of evidence to support that the fetus itself is involved in initiating signals of parturition, such as the secretion of hormones (cortisol, estrogen, placental corticotropin-releasing hormone (CRH)) and surfactant protein A (SP-A) [127]. The fetal adrenal gland is responsible for fetal cortisol production. Notably, placental CRH secretion may provide the first signal for cortisol stimulation [130]. Dysregulation or early activation of these processes may be implicated in the pathogenesis of PTB. Interestingly, SP-A, a major lung surfactant protein secreted by the maturing fetal lung when the capacity to sustain air breathing is developed, was identified in mice as a signal for parturition through its action on fetal macrophages [131]. SP-A binds to TLR4 on human and mouse macrophages and causes TLR4-dependent inflammatory cytokine expression [132]. SP-A was detected in the amniotic fluid at term pregnancy and stimulated IL1β and NF-κb expression in amniotic fluid–derived macrophages in vitro. Intra-amniotic injection of SP-A in mice additionally caused preterm delivery within 24 h [131]. Expression of steroid receptor coactivators SRC-1 and SRC-2, regulators of SP-A transcription, was determined to be key for the initiation of labor, and additionally key for the expression of PAF and lysophosphatidylcholine acyltransferase-1 (LPCAT1), which catalyzes the synthesis of PAF, in the fetal lung. As discussed, PAF initiates TLR signaling in mice and administration of PAF causes PTB [80, 109]. However, whether pathological activation of PAF signaling in PTB arises from the fetus is currently unknown.

In addition to fetal hormone- and SP-A-dependent modulation of labor and PTB, the fetal immune system has long been recognized to also have involvement in preterm birth [133]. The theory that the fetal immune system is immature has been robustly disproven, as neonatal T cells have the potential for activation [31, 54, 134]. Additionally, qualitative distinctions of immune responses between neonates and adults are in place. These include CD71^+^ erythroid cells, which are uniquely present in neonates and have been proposed to suppress microbial colonization-related inflammation. Also, IL8 (CXCL8) drives major T cell effector function in human newborns, as it allows for the activation of antimicrobial neutrophils and γδ T cells [135]. Importantly, these mechanisms may already instruct fetal immunity prior to birth during pregnancy and their dysregulation could be involved in the pathogenesis of PTB [53, 54, 134].

Interestingly, fetal macrophages appear to play a key role in initiating inflammatory cascade at parturition, as amniotic fluid fetal macrophages were shown to migrate to the uterus at term in response to increasing SP-A levels and engage with via TLR2 signaling [82, 131]. In neonates, polymorphic TLR4 and TLR2 alleles are associated with prematurity [98, 99]. Therefore, TLR signaling in fetal macrophages may be important in the pathogenesis of PTB. However, the phenotype of fetal macrophages seems to be tightly controlled to avoid inappropriate inflammation that may cause early activation of the inflammatory cascade leading to parturition [134] and failure of this control may result in an increased risk for PTB.

Insights arising from comparative cord blood analyses revealed distinct differences between term and preterm born infants [54]. Preterm cord blood was characterized by higher concentrations of inflammatory cytokines, increased activation of dendritic cells, and central memory (CCR7^+^CD45RA^−^) T cells with a Th1 phenotype. However, it needs to be confirmed if these differences are involved in the pathogenesis of PTB, or merely result from the less advanced immune ontogeny in preterm born neonates.

Emerging data arising from humans and mice also indicate that the fetal environment in PTB is also characterized by inflammation, along with the priming of fetal T cells against maternal antigens [54]. Activated fetal T cells are also found in the amniotic cavity and are increased in cases with chorioamnionitis [53]. Future studies are needed to confirm if fetal inflammation is the cause of the maternal inflammation and related PTB, or the consequence of the maternal spill-over of inflammatory cytokines.

The above-mentioned population of immunosuppressive CD71^+^ erythroid cells, which allows for microbial colonization upon birth, may play a functional role before birth through constraining fetal T cell activation. This is supported by observations upon depletion of CD71^+^ cells in splenocytes of neonatal mice, which caused robust cytokine production upon stimulation in vitro [134, 136].

## The potential role of FMC and MMC in PTB

The earlier introduced population of FMC cells may too represent a pathway through which the fetus modulates the time of labor. FMC, which increase over the course of gestation and peaking at term [32, 34], may be a key source of antigen that drives the accumulation of maternal-fetal antigen-specific Treg cells in pregnancy, hereby mediating fetal-maternal tolerance and maintenance of pregnancy [31]. Whether dysregulated FMC, e.g., resulting from fetal inflammation, are implicated in PTB is currently unknown. When FMCs were measured in maternal blood of term and PTL pregnancies, no quantitative differences were found [54], and insight on altered functions are still unknown.

As detectable amounts of MMC cells are transferred across the placenta to the fetus during pregnancy, there is a possibility for these cells to play a role in initiating the timing of birth. Indeed, increased MMC with T or B cells or myeloid cell phenotypes were found in mouse fetuses from mothers mated allogeneically and treated with LPS in late gestation [69]. In a human study, increased fetal DC and T cell activation in cord blood of preterm infants was associated with an increase in MMCs compared with term cord blood. A correlation between MMCs and central memory (CM) T cells was observed, leading to the hypothesis that the MMCs could be the source of maternal antigens to prime the alloreactive fetal T cells that are implicated in the induction of PTL [54]. However, as causality has not been shown, it needs to be addressed in future studies whether maternal cells trafficking to the fetus are the actual drivers of preterm birth and which mechanism is driving this phenomenon. One study addressed the impact of MMC cells on reproductive outcome in mice and describes that T cells specific for the NIMA on MMC mitigate reproductive fitness in the next generation [36]. Therefore, MMC cell transmission during gestation may play a role in the health and pregnancy trajectory of the subsequent offspring, including modulating their susceptibility to pregnancy complications such as PTB. However, this benefit may be restricted to pregnancies of the female filial generation where the paternal antigen specificity is overlapping between mating partner and father and future studies are needed to confirm this reproductive advantage irrespective of the parental antigen specificity. A study in humans also addressed the topic of MMC-mediated reproductive outcome and could identify that the presence of MMC cells from a woman’s own mother is associated with healthy pregnancy outcome, whereas no MMC cells could be detected in women diagnosed with preeclampsia, albeit specificity to NIMA could not be addressed [137]. To date, the role of cross-generational MMC has not yet been measured in women with PTL. However, this is an interesting prospect since women born preterm have an increased risk for PTB themselves [138].

## Non-cellular mediators and their potential role in PTB: fetal cfRNA, cfDNA, and exosomes

Fetal cell-free (cf)DNA has been investigated as a potential trigger of PTB. It is well known that fetal cfDNA, which increases steadily throughout normal pregnancy, is associated with labor [139]. cfDNA originates from the placenta via cell death and may act as a DAMP to initiate signals of labor [105]. Fetal cfDNA binds to TLR9 and induces NF-κb-dependent inflammatory effects [140]. Recently, several cfRNA transcripts, which have also been associated with immune, placental, or developmental function, can predict gestational age with comparable accuracy to ultrasound. Interestingly, cfRNA transcripts are enriched in women who delivered preterm [38]. The functional significance of these RNA transcripts in the pathogenesis of PTB is currently unknown. While they may represent novel biomarkers for the detection of PTB, understanding the molecular basis for their upregulation will be important for understanding the root causes of PTB.

Similarly, placental exosomes are detected at increased concentrations and have distinct compositions in pregnancy complications such as preeclampsia, IUGR, and PTB [43, 141]. Exosomes may mediate inflammatory signaling between fetal and maternal compartments in response to signals such as infection and stress. Exosomes are also detected in amniotic fluid and may play a role in labor induction, providing inflammatory signaling molecules [141]. Exosomes identified in human amniotic fluid showed distinct protein profiles in spontaneous PTB [141] and were shown to be enriched for inflammatory cytokines in women with intra-amniotic inflammation [50, 110]. Notably, DAMPs contained in fetal exosomes are released into maternal tissues and can induce inflammatory signaling pathways [102, 142].

Maternal exosomes must also be considered in initiating parturition. Human trophoblast explants internalize macrophage-derived exosomes, inducing secretion of pro-inflammatory cytokines IL6, IL8, IL10, and IL12 [49]. As macrophages infected with intracellular bacteria release PAMP-containing exosomes, which activate naïve T cells and macrophages [143, 144], this may provide a mechanism for the induction of decidual inflammation in infection-related PTB. Thus, macrophage-derived exosomes may traffic to and be taken up by the placenta to deliver key signals facilitating immunomodulatory pathways of parturition. Whether exosomes containing DAMPs are also delivered to the placenta in the case of sterile inflammation in term and preterm pregnancies is currently unknown.

## Clinical developments aiming to identify biomarker and therapeutic avenues

**Fig. 4:**
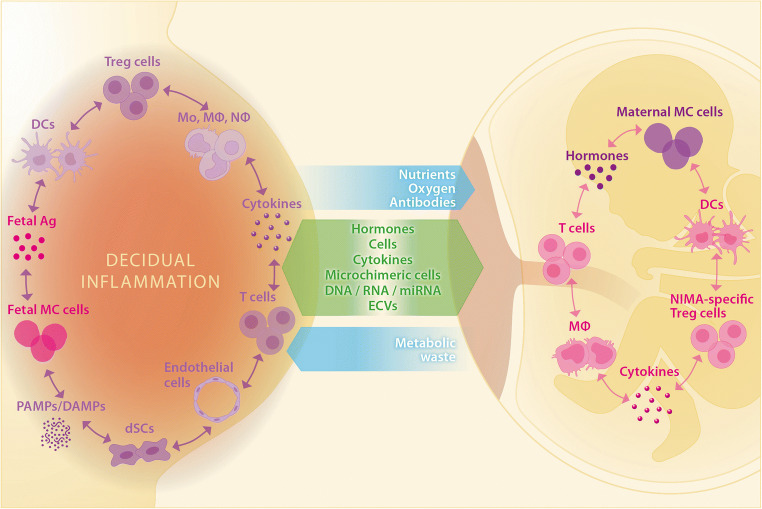
Fetal-maternal crosstalk is dysregulated in preterm versus normal pregnancy. Fetal-maternal crosstalk is essential for the progression of normal pregnancy. Via the placenta, nutrients, oxygen, and maternal antibodies are transferred to the fetus, and fetal metabolic waste is transferred to the mother. In addition, bidirectional transfer of key signaling molecules and cells occurs, such as the transfer of hormones, cytokines, nucleic acids, extracellular vesicles (ECVs), and microchimeric fetal and maternal cells. These molecules and cells influence both maternal and fetal immune environments. In the fetus, interactions between fetal immune cells and maternal MC cells induce fetal T cell tolerance to maternal antigens. As the fetus matures, the fetal lung and adrenal gland signal for parturition through fetal macrophages, which drive inflammation in the amniotic cavity. Approaching parturition, the decidual immune environment reflects interactions between various cells including macrophages, monocytes and T cells, and decidual endothelial and stromal cells. Decreased Treg cell activity may result in activation of fetal antigen-specific Teff cells. Fetal MC cells may provide a source of fetal antigen in the decidua. These cells respond to increasing inflammatory signals via PAMPs, DAMPs, and cytokines drive an inflammatory immune response that leads to the induction of labor. In preterm labor, various components of fetal-maternal crosstalk can be dysregulated, leading to premature activation of the inflammatory pathways off labor. This may occur due to a breakdown in fetal-maternal tolerance, whereby maternal T cells are activated towards fetal antigens, or fetal T cells are activated against maternal antigens

PTB is the leading cause of morbidity and mortality worldwide. Despite decades of research on the topic, the underlying etiology of PTB is still largely unknown. Multiple fetal-maternal signaling pathways exist and provide key communication between mother and fetus during pregnancy. This complexity clearly hampers the identification of the multilayered processes implicated in the immunopathogenesis of PTB (Fig. 4).

To date, therapeutic options for PTB are sparse. Current treatments to prevent suspected preterm birth include P4 supplementation and progestin prophylaxis. Other studies aimed at PTB prevention by targeting immune mediators. Here, the TLR4-antagonist (+)-naloxone, which is the non-opioid isomer of the opioid receptor antagonist (−)-naloxone, was tested in mice, where it suppressed the inflammatory cascade and prevent preterm birth [78]. Another immune-based therapeutic approach preventing PTB in mice targeted IL1β by using a novel IL1 receptor biased ligand [64]. Given the importance of IL10 mediating anti-inflammatory actions in the decidua, boosting IL10 in the decidua may represent one future strategy to combat PTB [67, 84]. The development of therapies aiming to increase Treg cells is also considered [145].

## Conclusion

Despite its essential nature, a detailed understanding of the molecular mechanisms underlying normal labor induction is only slowly emerging. PTB is a syndrome hypothesized to result from pathological activation of the normal physiological processes that lead to labor [7]. Yet most of our understanding of the pathways involved in labor originates from studies investigating single pathways or mediators by comparing preterm and term pregnancies in humans and various animal models. As multiomic assessments are now underway to evaluate the plausibility of immunological findings [146], the power of biological signatures predictive of preterm birth may soon be within reach and will inform novel approaches of early prediction and therapeutic interventions. Hence, understanding of molecular mechanisms will likely experience rapid developments in the near future, hereby enabling the identification of women at risk and the development of early prevention strategies of PTB.

## References

[1] Blencowe H, Cousens S, Chou D, Oestergaard M, Say L, Moller AB, Kinney M, Lawn J (2013). Born too soon: the global epidemiology of 15 million preterm births. Reprod Health.

[2] Markopoulou P, Papanikolaou E, Analytis A, Zoumakis E, Siahanidou T (2019). Preterm birth as a risk factor for metabolic syndrome and cardiovascular disease in adult life: a systematic review and meta-analysis. J Pediatr.

[3] Arpino C, Compagnone E, Montanaro M, Cacciatore D, Luca A, Cerulli A, Girolamo S, Curatolo P (2010). Preterm birth and neurodevelopmental outcome: a review. Childs Nerv Syst.

[4] Goedicke-Fritz S, Härtel C, Krasteva-Christ G, Kopp MV, Meyer S, Zemlin M (2017) Preterm birth affects the risk of developing immune-mediated diseases. Front Immunol 8(1266). 10.3389/fimmu.2017.01266 10.3389/fimmu.2017.01266 PMC5640887 29062316

[5] Sonnenschein-van der Voort AM, Arends LR, de Jongste JC, Annesi-Maesano I, Arshad SH, Barros H, Basterrechea M, Bisgaard H, Chatzi L, Corpeleijn E, Correia S, Craig LC, Devereux G, Dogaru C, Dostal M, Duchen K, Eggesbo M, van der Ent CK, Fantini MP, Forastiere F, Frey U, Gehring U, Gori D, van der Gugten AC, Hanke W, Henderson AJ, Heude B, Iniguez C, Inskip HM, Keil T, Kelleher CC, Kogevinas M, Kreiner-Moller E, Kuehni CE, Kupers LK, Lancz K, Larsen PS, Lau S, Ludvigsson J, Mommers M, Nybo Andersen AM, Palkovicova L, Pike KC, Pizzi C, Polanska K, Porta D, Richiardi L, Roberts G, Schmidt A, Sram RJ, Sunyer J, Thijs C, Torrent M, Viljoen K, Wijga AH, Vrijheid M, Jaddoe VW, Duijts L (2014). Preterm birth, infant weight gain, and childhood asthma risk: a meta-analysis of 147,000 European children. J Allergy Clin Immunol.

[6] Moster D, Lie RT, Markestad T (2008). Long-term medical and social consequences of preterm birth. N Engl J Med.

[7] Romero R, Dey SK, Fisher SJ (2014). Preterm labor: one syndrome, many causes. Science.

[8] Purisch SE, Gyamfi-Bannerman C (2017). Epidemiology of preterm birth. Semin Perinatol.

[9] Zhang G, Feenstra B, Bacelis J, Liu X, Muglia LM, Juodakis J, Miller DE, Litterman N, Jiang PP, Russell L, Hinds DA, Hu Y, Weirauch MT, Chen X, Chavan AR, Wagner GP, Pavlicev M, Nnamani MC, Maziarz J, Karjalainen MK, Ramet M, Sengpiel V, Geller F, Boyd HA, Palotie A, Momany A, Bedell B, Ryckman KK, Huusko JM, Forney CR, Kottyan LC, Hallman M, Teramo K, Nohr EA, Davey Smith G, Melbye M, Jacobsson B, Muglia LJ (2017). Genetic associations with gestational duration and spontaneous preterm birth. N Engl J Med.

[10] Norwitz ER, Bonney EA, Snegovskikh VV, Williams MA, Phillippe M, Park JS, Abrahams VM (2015). Molecular regulation of parturition: the role of the decidual clock. Cold Spring Harb Perspect Med.

[11] Romero R, Espinoza J, Goncalves LF, Kusanovic JP, Friel LA, Nien JK (2006). Inflammation in preterm and term labour and delivery. Semin Fetal Neonatal Med.

[12] Mohamed SA, Thota C, Browne PC, Diamond MP, Al-Hendy A (2014). Why is preterm birth stubbornly higher in African-Americans?. Obstet Gynecol Int J.

[13] Meuleman T, Lashley LELO, Dekkers OM, van Lith JMM, Claas FHJ, Bloemenkamp KWM (2015). HLA associations and HLA sharing in recurrent miscarriage: a systematic review and meta-analysis. Hum Immunol.

[14] Robertson SA, Moldenhauer LM (2014). Immunological determinants of implantation success. Int J Dev Biol.

[15] Arck PC, Hecher K (2013). Fetomaternal immune cross-talk and its consequences for maternal and offspring’s health. Nat Med.

[16] Deshmukh H, Way SS (2019). Immunological basis for recurrent fetal loss and pregnancy complications. Annu Rev Pathol.

[17] Robertson SA, Care AS, Moldenhauer LM (2018). Regulatory T cells in embryo implantation and the immune response to pregnancy. J Clin Invest.

[18] Jiang TT, Chaturvedi V, Ertelt JM, Kinder JM, Clark DR, Valent AM, Xin L, Way SS (2014). Regulatory T cells: new keys for further unlocking the enigma of fetal tolerance and pregnancy complications. J Immunol.

[19] Trowsdale J, Betz AG (2006). Mother’s little helpers: mechanisms of maternal-fetal tolerance. Nat Immunol.

[20] Ferreira LMR, Meissner TB, Tilburgs T, Strominger JL (2017). HLA-G: at the interface of maternal-fetal tolerance. Trends Immunol.

[21] Jones RL, Stoikos C, Findlay JK, Salamonsen LA (2006). TGF-beta superfamily expression and actions in the endometrium and placenta. Reproduction.

[22] Guerin LR, Moldenhauer LM, Prins JR, Bromfield JJ, Hayball JD, Robertson SA (2011). Seminal fluid regulates accumulation of FOXP3+ regulatory T cells in the preimplantation mouse uterus through expanding the FOXP3+ cell pool and CCL19-mediated recruitment. Biol Reprod.

[23] Aluvihare VR, Kallikourdis M, Betz AG (2004). Regulatory T cells mediate maternal tolerance to the fetus. Nat Immunol.

[24] Samstein RM, Josefowicz SZ, Arvey A, Treuting PM, Rudensky AY (2012). Extrathymic generation of regulatory T cells in placental mammals mitigates maternal-fetal conflict. Cell.

[25] Rowe JH, Ertelt JM, Xin L, Way SS (2012). Pregnancy imprints regulatory memory that sustains anergy to fetal antigen. Nature.

[26] Chen T, Darrasse-Jeze G, Bergot AS, Courau T, Churlaud G, Valdivia K, Strominger JL, Ruocco MG, Chaouat G, Klatzmann D (2013). Self-specific memory regulatory T cells protect embryos at implantation in mice. J Immunol.

[27] Moldenhauer LM, Diener KR, Thring DM, Brown MP, Hayball JD, Robertson SA (2009). Cross-presentation of male seminal fluid antigens elicits T cell activation to initiate the female immune response to pregnancy. J Immunol.

[28] Fallarino F, Grohmann U, Hwang KW, Orabona C, Vacca C, Bianchi R, Belladonna ML, Fioretti MC, Alegre M-L, Puccetti P (2003). Modulation of tryptophan catabolism by regulatory T cells. Nat Immunol.

[29] Kalekar LA, Schmiel SE, Nandiwada SL, Lam WY, Barsness LO, Zhang N, Stritesky GL, Malhotra D, Pauken KE, Linehan JL, O’Sullivan MG, Fife BT, Hogquist KA, Jenkins MK, Mueller DL (2016). CD4(+) T cell anergy prevents autoimmunity and generates regulatory T cell precursors. Nat Immunol.

[30] Svensson-Arvelund J, Mehta RB, Lindau R, Mirrasekhian E, Rodriguez-Martinez H, Berg G, Lash GE, Jenmalm MC, Ernerudh J (2015). The human fetal placenta promotes tolerance against the semiallogeneic fetus by inducing regulatory T cells and homeostatic M2 macrophages. J Immunol.

[31] Kinder JM, Stelzer IA, Arck PC, Way SS (2017). Immunological implications of pregnancy-induced microchimerism. Nat Rev Immunol.

[32] Ariga H, Ohto H, Busch MP, Imamura S, Watson R, Reed W, Lee TH (2001). Kinetics of fetal cellular and cell-free DNA in the maternal circulation during and after pregnancy: implications for noninvasive prenatal diagnosis. Transfusion.

[33] Walknowska J, Conte FA, Grumbach MM (1969). Practical and theoretical implications of fetal-maternal lymphocyte transfer. Lancet.

[34] Fujiki Y, Johnson KL, Tighiouart H, Peter I, Bianchi DW (2008). Fetomaternal trafficking in the mouse increases as delivery approaches and is highest in the maternal lung. Biol Reprod.

[35] Mold JE, Michaelsson J, Burt TD, Muench MO, Beckerman KP, Busch MP, Lee TH, Nixon DF, McCune JM (2008). Maternal alloantigens promote the development of tolerogenic fetal regulatory T cells in utero. Science.

[36] Kinder JM, Jiang TT, Ertelt JM, Xin L, Strong BS, Shaaban AF, Way SS (2015). Cross-generational reproductive fitness enforced by microchimeric maternal cells. Cell.

[37] Koh W, Pan W, Gawad C, Fan HC, Kerchner GA, Wyss-Coray T, Blumenfeld YJ, El-Sayed YY, Quake SR (2014). Noninvasive in vivo monitoring of tissue-specific global gene expression in humans. Proc Natl Acad Sci.

[38] Ngo TTM, Moufarrej MN, Rasmussen MH, Camunas-Soler J, Pan W, Okamoto J, Neff NF, Liu K, Wong RJ, Downes K, Tibshirani R, Shaw GM, Skotte L, Stevenson DK, Biggio JR, Elovitz MA, Melbye M, Quake SR (2018). Noninvasive blood tests for fetal development predict gestational age and preterm delivery. Science.

[39] Poon LLM, Leung TN, Lau TK, Lo YMD (2000). Presence of fetal RNA in maternal plasma. Clin Chem.

[40] Maron JL, Johnson KL, Slonim D, Lai CQ, Ramoni M, Alterovitz G, Jarrah Z, Yang Z, Bianchi DW (2007). Gene expression analysis in pregnant women and their infants identifies unique fetal biomarkers that circulate in maternal blood. J Clin Invest.

[41] van Wijk IJ, de Hoon AC, Jurhawan R, Tjoa ML, Griffioen S, Mulders MAM, van Vugt JMG, Oudejans CBM (2000). Detection of apoptotic fetal cells in plasma of pregnant women. Clin Chem.

[42] Lo YM, Corbetta N, Chamberlain PF, Rai V, Sargent IL, Redman CW, Wainscoat JS (1997). Presence of fetal DNA in maternal plasma and serum. Lancet.

[43] Mitchell MD, Peiris HN, Kobayashi M, Koh YQ, Duncombe G, Illanes SE, Rice GE, Salomon C (2015). Placental exosomes in normal and complicated pregnancy. Am J Obstet Gynecol.

[44] Sarker S, Scholz-Romero K, Perez A, Illanes SE, Mitchell MD, Rice GE, Salomon C (2014). Placenta-derived exosomes continuously increase in maternal circulation over the first trimester of pregnancy. J Transl Med.

[45] Salomon C, Torres MJ, Kobayashi M, Scholz-Romero K, Sobrevia L, Dobierzewska A, Illanes SE, Mitchell MD, Rice GE (2014). A gestational profile of placental exosomes in maternal plasma and their effects on endothelial cell migration. PLoS One.

[46] Desrochers LM, Bordeleau F, Reinhart-King CA, Cerione RA, Antonyak MA (2016). Microvesicles provide a mechanism for intercellular communication by embryonic stem cells during embryo implantation. Nat Commun.

[47] Luo SS, Ishibashi O, Ishikawa G, Ishikawa T, Katayama A, Mishima T, Takizawa T, Shigihara T, Goto T, Izumi A, Ohkuchi A, Matsubara S, Takeshita T, Takizawa T (2009). Human villous trophoblasts express and secrete placenta-specific microRNAs into maternal circulation via exosomes. Biol Reprod.

[48] Ouyang Y, Mouillet JF, Coyne CB, Sadovsky Y (2014). Review: placenta-specific microRNAs in exosomes - good things come in nano-packages. Placenta.

[49] Holder B, Jones T, Sancho Shimizu V, Rice TF, Donaldson B, Bouqueau M, Forbes K, Kampmann B (2016). Macrophage exosomes induce placental inflammatory cytokines: a novel mode of maternal-placental messaging. Traffic.

[50] Romero R, Grivel JC, Tarca AL, Chaemsaithong P, Xu Z, Fitzgerald W, Hassan SS, Chaiworapongsa T, Margolis L (2015). Evidence of perturbations of the cytokine network in preterm labor. Am J Obstet Gynecol.

[51] Soto E, Romero R, Richani K, Yoon BH, Chaiworapongsa T, Vaisbuch E, Mittal P, Erez O, Gotsch F, Mazor M, Kusanovic JP (2009). Evidence for complement activation in the amniotic fluid of women with spontaneous preterm labor and intra-amniotic infection. J Matern Fetal Neonatal Med.

[52] Schober L, Radnai D, Schmitt E, Mahnke K, Sohn C, Steinborn A (2012). Term and preterm labor: decreased suppressive activity and changes in composition of the regulatory T-cell pool. Immunol Cell Biol.

[53] Gomez-Lopez N, Romero R, Xu Y, Miller D, Arenas-Hernandez M, Garcia-Flores V, Panaitescu B, Galaz J, Hsu CD, Para R, Berry SM (2019). Fetal T cell activation in the amniotic cavity during preterm labor: a potential mechanism for a subset of idiopathic preterm birth. J Immunol.

[54] Frascoli M, Coniglio L, Witt R, Jeanty C, Fleck-Derderian S, Myers DE, Lee TH, Keating S, Busch MP, Norris PJ, Tang Q, Cruz G, Barcellos LF, Gomez-Lopez N, Romero R, MacKenzie TC (2018) Alloreactive fetal T cells promote uterine contractility in preterm labor via IFN-gamma and TNF-alpha. Sci Transl Med 10(438). 10.1126/scitranslmed.aan2263 10.1126/scitranslmed.aan2263 PMC6449052 29695455

[55] Elovitz MA, Mrinalini C (2004). Animal models of preterm birth. Trends Endocrinol Metab.

[56] Brosens I, Pijnenborg R, Vercruysse L, Romero R (2011). The "great obstetrical syndromes" are associated with disorders of deep placentation. Am J Obstet Gynecol.

[57] Romero R, Kusanovic JP, Chaiworapongsa T, Hassan SS (2011). Placental bed disorders in preterm labor, preterm PROM, spontaneous abortion and abruptio placentae. Best Pract Res Clin Obstet Gynaecol.

[58] Cao-Lei L, Laplante DP, King S (2016). Prenatal maternal stress and epigenetics: review of the human research. Curr Mol Biol Rep.

[59] Bashiri A, Halper KI, Orvieto R (2018). Recurrent implantation failure-update overview on etiology, diagnosis, treatment and future directions. Reprod Biol Endocrinol.

[60] Fettweis JM, Serrano MG, Brooks JP, Edwards DJ, Girerd PH, Parikh HI, Huang B, Arodz TJ, Edupuganti L, Glascock AL, Xu J, Jimenez NR, Vivadelli SC, Fong SS, Sheth NU, Jean S, Lee V, Bokhari YA, Lara AM, Mistry SD, Duckworth RA, Bradley SP, Koparde VN, Orenda XV, Milton SH, Rozycki SK, Matveyev AV, Wright ML, Huzurbazar SV, Jackson EM, Smirnova E, Korlach J, Tsai Y-C, Dickinson MR, Brooks JL, Drake JI, Chaffin DO, Sexton AL, Gravett MG, Rubens CE, Wijesooriya NR, Hendricks-Muñoz KD, Jefferson KK, Strauss JF, Buck GA (2019). The vaginal microbiome and preterm birth. Nat Med.

[61] Serrano MG, Parikh HI, Brooks JP, Edwards DJ, Arodz TJ, Edupuganti L, Huang B, Girerd PH, Bokhari YA, Bradley SP, Brooks JL, Dickinson MR, Drake JI, Duckworth RA, Fong SS, Glascock AL, Jean S, Jimenez NR, Khoury J, Koparde VN, Lara AM, Lee V, Matveyev AV, Milton SH, Mistry SD, Rozycki SK, Sheth NU, Smirnova E, Vivadelli SC, Wijesooriya NR, Xu J, Xu P, Chaffin DO, Sexton AL, Gravett MG, Rubens CE, Hendricks-Muñoz KD, Jefferson KK, Strauss JF, Fettweis JM, Buck GA (2019). Racioethnic diversity in the dynamics of the vaginal microbiome during pregnancy. Nat Med.

[62] Gargano JW, Holzman CB, Senagore PK, Reuss ML, Pathak DR, Williams MA, Fisher R (2010). Evidence of placental haemorrhage and preterm delivery. BJOG Int J Obstet Gynaecol.

[63] Cappelletti M, Presicce P, Lawson MJ, Chaturvedi V, Stankiewicz TE, Vanoni S, Harley ITW, McAlees JW, Giles DA, Moreno-Fernandez ME, Rueda CM, Senthamaraikannan P, Sun X, Karns R, Hoebe K, Janssen EM, Karp CL, Hildeman DA, Hogan SP, Kallapur SG, Chougnet CA, Way SS, Divanovic S (2017) Type I interferons regulate susceptibility to inflammation-induced preterm birth. JCI Insight 2(5). 10.1172/jci.insight.91288 10.1172/jci.insight.91288 PMC5333966 28289719

[64] Nadeau-Vallee M, Quiniou C, Palacios J, Hou X, Erfani A, Madaan A, Sanchez M, Leimert K, Boudreault A, Duhamel F, Rivera JC, Zhu T, Noueihed B, Robertson SA, Ni X, Olson DM, Lubell W, Girard S, Chemtob S (2015). Novel noncompetitive IL-1 receptor-biased ligand prevents infection- and inflammation-induced preterm birth. J Immunol.

[65] Robertson SA, Christiaens I, Dorian CL, Zaragoza DB, Care AS, Banks AM, Olson DM (2010). Interleukin-6 is an essential determinant of on-time parturition in the mouse. Endocrinology.

[66] Slutsky R, Romero R, Xu Y, Galaz J, Miller D, Done B, Tarca AL, Gregor S, Hassan SS, Leng Y, Gomez-Lopez N (2019). Exhausted and senescent T cells at the maternal-fetal Interface in preterm and term labor. J Immunol Res.

[67] Robertson SA, Skinner RJ, Care AS (2006). Essential role for IL-10 in resistance to lipopolysaccharide-induced preterm labor in mice. J Immunol.

[68] St Louis D, Romero R, Plazyo O, Arenas-Hernandez M, Panaitescu B, Xu Y, Milovic T, Xu Z, Bhatti G, Mi QS, Drewlo S, Tarca AL, Hassan SS, Gomez-Lopez N (2016). Invariant NKT cell activation induces late preterm birth that is attenuated by rosiglitazone. J Immunol.

[69] Wegorzewska M, Le T, Tang Q, MacKenzie TC (2014). Increased maternal T cell microchimerism in the allogeneic fetus during LPS-induced preterm labor in mice. Chimerism.

[70] Wahid HH, Dorian CL, Chin PY, Hutchinson MR, Rice KC, Olson DM, Moldenhauer LM, Robertson SA (2015). Toll-like receptor 4 is an essential upstream regulator of on-time parturition and perinatal viability in mice. Endocrinology.

[71] Filipovich Y, Lu SJ, Akira S, Hirsch E (2009). The adaptor protein MyD88 is essential for E coli-induced preterm delivery in mice. Am J Obstet Gynecol.

[72] Romero R, Tartakovsky B (1992). The natural interleukin-1 receptor antagonist prevents interleukin-1-induced preterm delivery in mice. Am J Obstet Gynecol.

[73] Arenas-Hernandez M, Romero R, Xu Y, Panaitescu B, Garcia-Flores V, Miller D, Ahn H, Done B, Hassan SS, Hsu CD, Tarca AL, Sanchez-Torres C, Gomez-Lopez N (2019). Effector and activated T cells induce preterm labor and birth that is prevented by treatment with progesterone. J Immunol.

[74] Boyson JE, Nagarkatti N, Nizam L, Exley MA, Strominger JL (2006). Gestation stage-dependent mechanisms of invariant natural killer T cell-mediated pregnancy loss. Proc Natl Acad Sci U S A.

[75] Sheller-Miller S, Trivedi J, Yellon SM, Menon R (2019). Exosomes cause preterm birth in mice: evidence for paracrine signaling in pregnancy. Sci Rep.

[76] Gomez-Lopez N, Romero R, Plazyo O, Panaitescu B, Furcron AE, Miller D, Roumayah T, Flom E, Hassan SS (2016). Intra-amniotic administration of HMGB1 induces spontaneous preterm labor and birth. Am J Reprod Immunol.

[77] Gonzalez JM, Franzke CW, Yang F, Romero R, Girardi G (2011). Complement activation triggers metalloproteinases release inducing cervical remodeling and preterm birth in mice. Am J Pathol.

[78] Chin PY, Dorian CL, Hutchinson MR, Olson DM, Rice KC, Moldenhauer LM, Robertson SA (2016). Novel toll-like receptor-4 antagonist (+)-naloxone protects mice from inflammation-induced preterm birth. Sci Rep.

[79] Bizargity P, Del Rio R, Phillippe M, Teuscher C, Bonney EA (2009). Resistance to lipopolysaccharide-induced preterm delivery mediated by regulatory T cell function in mice. Biol Reprod.

[80] Agrawal V, Jaiswal MK, Ilievski V, Beaman KD, Jilling T, Hirsch E (2014). Platelet-activating factor: a role in preterm delivery and an essential interaction with toll-like receptor signaling in mice. Biol Reprod.

[81] Gao L, Rabbitt EH, Condon JC, Renthal NE, Johnston JM, Mitsche MA, Chambon P, Xu J, O’Malley BW, Mendelson CR (2015). Steroid receptor coactivators 1 and 2 mediate fetal-to-maternal signaling that initiates parturition. J Clin Invest.

[82] Montalbano AP, Hawgood S, Mendelson CR (2013). Mice deficient in surfactant protein A (SP-A) and SP-D or in TLR2 manifest delayed parturition and decreased expression of inflammatory and contractile genes. Endocrinology.

[83] Mizoguchi M, Ishida Y, Nosaka M, Kimura A, Kuninaka Y, Yahata T, Nanjo S, Toujima S, Minami S, Ino K, Mukaida N, Kondo T (2018). Prevention of lipopolysaccharide-induced preterm labor by the lack of CX3CL1-CX3CR1 interaction in mice. PLoS One.

[84] Deng W, Yuan J, Cha J, Sun X, Bartos A, Yagita H, Hirota Y, Dey SK (2019). Endothelial cells in the decidual bed are potential therapeutic targets for preterm birth prevention. Cell Rep.

[85] Hirota Y, Cha J, Yoshie M, Daikoku T, Dey SK (2011). Heightened uterine mammalian target of rapamycin complex 1 (mTORC1) signaling provokes preterm birth in mice. Proc Natl Acad Sci U S A.

[86] Cha J, Bartos A, Egashira M, Haraguchi H, Saito-Fujita T, Leishman E, Bradshaw H, Dey SK, Hirota Y (2013). Combinatory approaches prevent preterm birth profoundly exacerbated by gene-environment interactions. J Clin Invest.

[87] Yoshida M, Takayanagi Y, Ichino-Yamashita A, Sato K, Sugimoto Y, Kimura T, Nishimori K (2019). Functional hierarchy of uterotonics required for successful parturition in mice. Endocrinology.

[88] McCarthy R, Martin-Fairey C, Sojka DK, Herzog ED, Jungheim ES, Stout MJ, Fay JC, Mahendroo M, Reese J, Herington JL, Plosa EJ, Shelton EL, England SK (2018). Mouse models of preterm birth: suggested assessment and reporting guidelines. Biol Reprod.

[89] Murray SA, Morgan JL, Kane C, Sharma Y, Heffner CS, Lake J, Donahue LR (2010). Mouse gestation length is genetically determined. PLoS One.

[90] Lindström TM, Bennett PR (2005). The role of nuclear factor kappa B in human labour. Reproduction.

[91] Kisielewicz A, Schaier M, Schmitt E, Hug F, Haensch GM, Meuer S, Zeier M, Sohn C, Steinborn A (2010). A distinct subset of HLA-DR+-regulatory T cells is involved in the induction of preterm labor during pregnancy and in the induction of organ rejection after transplantation. Clin Immunol.

[92] Shah NM, Edey LF, Imami N, Johnson MR (2020). Human labour is associated with altered regulatory T cell function and maternal immune activation. Clin Exp Immunol.

[93] Gomez-Lopez N, Vega-Sanchez R, Castillo-Castrejon M, Romero R, Cubeiro-Arreola K, Vadillo-Ortega F (2013). Evidence for a role for the adaptive immune response in human term parturition. Am J Reprod Immunol.

[94] Boyson JE, Rybalov B, Koopman LA, Exley M, Balk SP, Racke FK, Schatz F, Masch R, Wilson SB, Strominger JL (2002). CD1d and invariant NKT cells at the human maternal-fetal interface. Proc Natl Acad Sci U S A.

[95] Li LP, Fang YC, Dong GF, Lin Y, Saito S (2012). Depletion of invariant NKT cells reduces inflammation-induced preterm delivery in mice. J Immunol.

[96] Liassides C, Papadopoulos A, Siristatidis C, Damoraki G, Liassidou A, Chrelias C, Kassanos D, Giamarellos-Bourboulis EJ (2019). Single nucleotide polymorphisms of toll-like receptor-4 and of autophagy-related gene 16 like-1 gene for predisposition of premature delivery: a prospective study. Medicine.

[97] Patni S, Wynen LP, Seager AL, Morgan G, White JO, Thornton CA (2009). Expression and activity of toll-like receptors 1–9 in the human term placenta and changes associated with labor at term. Biol Reprod.

[98] Krediet TG, Wiertsema SP, Vossers MJ, Hoeks SBEA, Fleer A, Ruven HJT, Rijkers GT (2007). Toll-like receptor 2 polymorphism is associated with preterm birth. Pediatr Res.

[99] Lorenz E, Hallman M, Marttila R, Haataja R, Schwartz DA (2002). Association between the Asp299Gly polymorphisms in the toll-like receptor 4 and premature births in the Finnish population. Pediatr Res.

[100] Romero R, Xu Y, Plazyo O, Chaemsaithong P, Chaiworapongsa T, Unkel R, Than NG, Chiang PJ, Dong Z, Xu Z, Tarca AL, Abrahams VM, Hassan SS, Yeo L, Gomez-Lopez N (2018). A role for the Inflammasome in spontaneous labor at term. Am J Reprod Immunol.

[101] Nadeau-Vallée M, Obari D, Palacios J, Brien M-È, Duval C, Chemtob S, Girard S (2016). Sterile inflammation and pregnancy complications: a review. Reproduction.

[102] Menon R (2019). Initiation of human parturition: signaling from senescent fetal tissues via extracellular vesicle mediated paracrine mechanism. Obstet Gynecol Sci.

[103] Romero R, Chaiworapongsa T, Alpay Savasan Z, Xu Y, Hussein Y, Dong Z, Kusanovic JP, Kim CJ, Hassan SS (2011). Damage-associated molecular patterns (DAMPs) in preterm labor with intact membranes and preterm PROM: a study of the alarmin HMGB1. J Matern Fetal Neonatal Med.

[104] Plazyo O, Romero R, Unkel R, Balancio A, Mial TN, Xu Y, Dong Z, Hassan SS, Gomez-Lopez N (2016) HMGB1 induces an inflammatory response in the chorioamniotic membranes that is partially mediated by the inflammasome1. Biol Reprod 95(6). 10.1095/biolreprod.116.144139 10.1095/biolreprod.116.144139 PMC5315428 27806943

[105] Boeckel SR, Davidson DJ, Norman JE, Stock SJ (2018). Cell-free fetal DNA and spontaneous preterm birth. Reproduction.

[106] Silver RK, Caplan MS, Kelly AM (1992). Amniotic fluid platelet-activating factor (PAF) is elevated in patients with tocolytic failure and preterm delivery. Prostaglandins.

[107] Hoffman DR, Romero R, Johnston JM (1990). Detection of platelet-activating factor in amniotic fluid of complicated pregnancies. Am J Obstet Gynecol.

[108] Zhu YP, Hoffman DR, Hwang SB, Miyaura S, Johnston JM (1991). Prolongation of parturition in the pregnant rat following treatment with a platelet activating factor receptor antagonist. Biol Reprod.

[109] Elovitz MA, Wang Z, Chien EK, Rychlik DF, Phillippe M (2003). A new model for inflammation-induced preterm birth: the role of platelet-activating factor and toll-like receptor-4. Am J Pathol.

[110] Bhatti G, Romero R, Rice GE, Fitzgerald W, Pacora P, Gomez-Lopez N, Kavdia M, Tarca AL, Margolis L (2020). Compartmentalized profiling of amniotic fluid cytokines in women with preterm labor. PLoS One.

[111] Bayar E, Bennett PR, Chan D, Sykes L, MacIntyre DA (2020) The pregnancy microbiome and preterm birth. Seminars in Immunopathology. 10.1007/s00281-020-00817-w 10.1007/s00281-020-00817-w PMC7508933 32797272

[112] Andrews WW, Goldenberg RL, Hauth JC, Cliver SP, Copper R, Conner M (2006). Interconceptional antibiotics to prevent spontaneous preterm birth: a randomized clinical trial. Am J Obstet Gynecol.

[113] Espinoza J, Erez O, Romero R (2006). Preconceptional antibiotic treatment to prevent preterm birth in women with a previous preterm delivery. Am J Obstet Gynecol.

[114] Chen C, Song X, Wei W, Zhong H, Dai J, Lan Z, Li F, Yu X, Feng Q, Wang Z, Xie H, Chen X, Zeng C, Wen B, Zeng L, Du H, Tang H, Xu C, Xia Y, Xia H, Yang H, Wang J, Wang J, Madsen L, Brix S, Kristiansen K, Xu X, Li J, Wu R, Jia H (2017). The microbiota continuum along the female reproductive tract and its relation to uterine-related diseases. Nat Commun.

[115] Moreno I, Codoñer FM, Vilella F, Valbuena D, Martinez-Blanch JF, Jimenez-Almazán J, Alonso R, Alamá P, Remohí J, Pellicer A, Ramon D, Simon C (2016). Evidence that the endometrial microbiota has an effect on implantation success or failure. Am J Obstet Gynecol.

[116] Aagaard K, Ma J, Antony KM, Ganu R, Petrosino J, Versalovic J (2014). The placenta harbors a unique microbiome. Sci Transl Med.

[117] de Goffau MC, Lager S, Sovio U, Gaccioli F, Cook E, Peacock SJ, Parkhill J, Charnock-Jones DS, Smith GCS (2019). Human placenta has no microbiome but can contain potential pathogens. Nature.

[118] Nadeem L, Shynlova O, Matysiak-Zablocki E, Mesiano S, Dong X, Lye S (2016). Molecular evidence of functional progesterone withdrawal in human myometrium. Nat Commun.

[119] MURR SM, STABENFELDT GH, BRADFORD GE, GESCHWIND II (1974). Plasma progesterone during pregnancy in the mouse1. Endocrinology.

[120] Piekorz RP, Gingras S, Hoffmeyer A, Ihle JN, Weinstein Y (2005). Regulation of progesterone levels during pregnancy and parturition by signal transducer and activator of transcription 5 and 20alpha-hydroxysteroid dehydrogenase. Mol Endocrinol.

[121] Dudley DJ, Branch DW, Edwin SS, Mitchell MD (1996). Induction of preterm birth in mice by RU486. Biol Reprod.

[122] Lee JH, Lydon JP, Kim CH (2012). Progesterone suppresses the mTOR pathway and promotes generation of induced regulatory T cells with increased stability. Eur J Immunol.

[123] Hierweger AM, Engler JB, Friese MA, Reichardt HM, Lydon J, DeMayo F, Mittrücker H-W, Arck PC (2019). Progesterone modulates the T-cell response via glucocorticoid receptor-dependent pathways. Am J Reprod Immunol.

[124] Thiele K, Hierweger AM, Riquelme JIA, Solano ME, Lydon JP, Arck PC (2019). Impaired progesterone-responsiveness of CD11c(+) dendritic cells affects the generation of CD4(+) regulatory T cells and is associated with intrauterine growth restriction in mice. Front Endocrinol (Lausanne).

[125] Care AS, Diener KR, Jasper MJ, Brown HM, Ingman WV, Robertson SA (2013). Macrophages regulate corpus luteum development during embryo implantation in mice. J Clin Invest.

[126] Hirsch E, Muhle R (2002). Intrauterine bacterial inoculation induces labor in the mouse by mechanisms other than progesterone withdrawal. Biol Reprod.

[127] Mendelson CR, Gao L, Montalbano AP (2019) Multifactorial regulation of myometrial contractility during pregnancy and parturition. Front Endocrinol 10(714). 10.3389/fendo.2019.00714 10.3389/fendo.2019.00714 PMC6823183 31708868

[128] Anamthathmakula P, Kyathanahalli C, Ingles J, Hassan SS, Condon JC, Jeyasuria P (2019). Estrogen receptor alpha isoform ERdelta7 in myometrium modulates uterine quiescence during pregnancy. EBioMedicine.

[129] Nancy P, Siewiera J, Rizzuto G, Tagliani E, Osokine I, Manandhar P, Dolgalev I, Clementi C, Tsirigos A, Erlebacher A (2018). H3K27me3 dynamics dictate evolving uterine states in pregnancy and parturition. J Clin Invest.

[130] Torricelli M, Giovannelli A, Leucci E, De Falco G, Reis FM, Imperatore A, Florio P, Petraglia F (2007). Labor (term and preterm) is associated with changes in the placental mRNA expression of corticotrophin-releasing factor. Reprod Sci.

[131] Condon JC, Jeyasuria P, Faust JM, Mendelson CR (2004). Surfactant protein secreted by the maturing mouse fetal lung acts as a hormone that signals the initiation of parturition. Proc Natl Acad Sci U S A.

[132] Guillot L, Balloy V, McCormack FX, Golenbock DT, Chignard M, Si-Tahar M (2002). Cutting edge: the immunostimulatory activity of the lung surfactant protein-A involves toll-like receptor 4. J Immunol.

[133] Berry SM, Romero R, Gomez R, Puder KS, Ghezzi F, Cotton DB, Bianchi DW (1995). Premature parturition is characterized by in utero activation of the fetal immune system. Am J Obstet Gynecol.

[134] Elahi S, Ertelt JM, Kinder JM, Jiang TT, Zhang X, Xin L, Chaturvedi V, Strong BS, Qualls JE, Steinbrecher KA, Kalfa TA, Shaaban AF, Way SS (2013). Immunosuppressive CD71+ erythroid cells compromise neonatal host defence against infection. Nature.

[135] Gibbons D, Fleming P, Virasami A, Michel M-L, Sebire NJ, Costeloe K, Carr R, Klein N, Hayday A (2014). Interleukin-8 (CXCL8) production is a signatory T cell effector function of human newborn infants. Nat Med.

[136] Delyea C, Bozorgmehr N, Koleva P, Dunsmore G, Shahbaz S, Huang V, Elahi S (2018). CD71(+) erythroid suppressor cells promote fetomaternal tolerance through arginase-2 and PDL-1. J Immunol.

[137] Gammill HS, Adams Waldorf KM, Aydelotte TM, Lucas J, Leisenring WM, Lambert NC, Nelson JL (2011). Pregnancy, microchimerism, and the maternal grandmother. PLoS One.

[138] Boyd HA, Poulsen G, Wohlfahrt J, Murray JC, Feenstra B, Melbye M (2009). Maternal contributions to preterm delivery. Am J Epidemiol.

[139] Lo Y, Zhang J, Leung T, Lau T, Chang A, Hjelm N (1999). Rapid clearance of fetal DNA from maternal plasma. Am J Hum Genet.

[140] Scharfe-Nugent A, Corr S, Carpenter S, Keogh L, Doyle B, Martin C, Fitzgerald K, Daly S, O’Leary J, O’Neill L (2012). TLR9 provokes inflammation in response to fetal DNA: mechanism for fetal loss in preterm birth and preeclampsia. J Immunol.

[141] Dixon CL, Sheller-Miller S, Saade GR, Fortunato SJ, Lai A, Palma C, Guanzon D, Salomon C, Menon R (2018). Amniotic fluid exosome proteomic profile exhibits unique pathways of term and preterm labor. Endocrinology.

[142] Sheller-Miller S, Urrabaz-Garza R, Saade G, Menon R (2017). Damage-associated molecular pattern markers HMGB1 and cell-free fetal telomere fragments in oxidative-stressed amnion epithelial cell-derived exosomes. J Reprod Immunol.

[143] Bhatnagar S, Shinagawa K, Castellino FJ, Schorey JS (2007). Exosomes released from macrophages infected with intracellular pathogens stimulate a proinflammatory response in vitro and in vivo. Blood.

[144] Giri PK, Schorey JS (2008). Exosomes derived from M. Bovis BCG infected macrophages activate antigen-specific CD4+ and CD8+ T cells in vitro and in vivo. PLoS One.

[145] Robertson SA, Green ES, Care AS, Moldenhauer LM, Prins JR, Hull ML, Barry SC, Dekker G (2019). Therapeutic potential of regulatory T cells in preeclampsia-opportunities and challenges. Front Immunol.

[146] Peterson LS, Stelzer IA, Tsai AS, Ghaemi MS, Han X, Ando K, Winn VD, Martinez NR, Contrepois K, Moufarrej MN, Quake S, Relman DA, Snyder MP, Shaw GM, Stevenson DK, Wong RJ, Arck P, Angst MS, Aghaeepour N, Gaudilliere B (2020) Multiomic immune clockworks of pregnancy. Semin Immunopathol. 10.1007/s00281-019-00772-1 10.1007/s00281-019-00772-1 PMC7508753 32020337

